# Preserving the intercostal nerves as a goal in thoracotomy

**DOI:** 10.1590/S1806-37132014000600013

**Published:** 2014

**Authors:** Roberto de Menezes Lyra

**Affiliations:** São Paulo Hospital for State Civil Servants, São Paulo, Brazil. São Paulo Hospital for State Civil Servants, São Paulo, Brazil

## To the Editor:

It is difficult to assess and quantify immediate postoperative pain clinically, which is why I enthusiastically read the article in which Marchetti-Filho et al.^(^ 1 ^)^ describe the role of intercostal nerve preservation in acute pain control after thoracotomy. I would like to comment on the technique for intercostal nerve preservation. 

Thoracic surgeons should certainly be able to deal with acute and chronic post-thoracotomy pain. In the past, neuropathic pain occurred in approximately 50% of patients, being generally mild or moderate. However, it is estimated to persist in 5% of cases, leading to disability. In such cases, three-dimensional reconstruction of CT scans of the chest has been used in order to assist in the decision of whether to perform intercostal neurolysis.^(^ 2 ^)^

Surgical techniques to prevent intercostal nerve injury should be validated and used in order to reduce the incidence of acute and chronic post-thoracotomy pain.^(^ 3 ^)^

After an extensive thoracotomy, the intercostal space should be opened slowly and gradually with the Finochietto retractor in order to avoid rib fractures. However, the amount of rib spreading can suddenly overcome the resistance of one or more ribs, causing one or more fractures. A rib fracture can be accompanied by neurovascular bundle injury at the upper and lower ribs. This can result in chronic neuralgia and paresthesia in the postoperative period. 

Rib spreading with the Finochietto retractor is traumatic because the neurovascular bundle at the upper rib is crushed and remains so for a long period of time. In order to avoid intercostal nerve compression by the Finochietto retractor, it has been proposed that an intercostal muscle flap be harvested before placement of the retractor.^(^ 4 ^)^

In the past, circumcostal suture placement for rib approximation was widely used for thoracotomy closure. However, it often caused neuropathic pain as a result of the suture material compressing the neurovascular bundle at the lower rib. 

The current intercostal nerve preservation strategy requires that care be taken when closing a thoracotomy. 

There are currently three thoracotomy closure techniques:

- transosteal or transcostal suture closure
- transperiosteal suture closure^(^ 5 ^)^
- pericostal suture closure, whereby sutures are placed in the virtual space between the periosteum and the neurovascular bundle^(^ 6 ^)^

Three interrupted sutures are generally placed in order to close an extensive thoracotomy. When a rib fracture occurs, one or more sutures are needed in order to stabilize and align the fracture fragments. Synthetic absorbable 1-0 polyglactin 910 suture should be used whenever possible. For increased suture strength, a double-loop, U-shaped suture (i.e., a double 1-0 suture) is generally recommended. 

Below, I describe the thoracotomy closure technique whereby sutures are placed in the virtual space between the periosteum and the neurovascular bundle at the lower rib. 

The surgical needle with absorbable 1-0 suture is passed into the thoracic cavity through a point above the upper edge of the rib cranial to the thoracotomy. The needle inside the thoracic cavity is retrieved with the needle holder, and the suture is pulled until it reaches half of its length. 

The needle goes into the thoracic cavity again and out of the chest wall through a point located very close to the entry point. A U-shaped suture (i.e., a double 1-0 suture) is thus obtained, the strength of which is greater. The needle is removed, and the suture ends are trimmed with straight Kelly forceps. 

The suture end inside the thoracic cavity is pulled out and looped around the Kelly forceps so that the double suture is halved. 

The procedure described above is performed for each of the three or four sutures required for thoracotomy closure, thus concluding the first step of the closure technique. 

**Figure 1 -:**
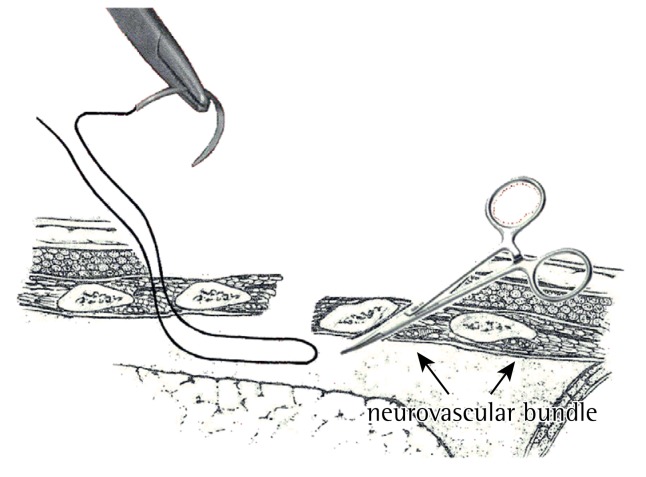
Preparation for thoracotomy closure. Blunt dissection is performed in the virtual space between the lower rib and the neurovascular bundle so that the suture material can pass through the chest wall without compressing the intercostal nerve during thoracotomy closure.

At the lower edge of the rib caudal to the thoracotomy, blunt dissection is performed with curved Halstead forceps so that the tip of the instrument enters the thoracic cavity (Figure 1). The dissection (extraperiosteal and pericostal dissection) is performed in the small, virtual space between the periosteum and the neurovascular bundle, thus preventing neurovascular bundle injury, the neurovascular bundle remaining outside of the suture attachment point. 

Minor bleeding from the blunt dissection should be managed so that hemostasis is achieved at this point. 

With the use of dissecting forceps, the suture end is led to the tips of the curved Halstead forceps, being clamped and pulled out of the thoracic cavity. This is done for all sutures passed through. 

Subsequently, each double suture is firmly tightened and tied with a surgeon's knot. Care must be taken to prevent the tied ribs from touching one another or from overlapping. After the remaining suture is cut and removed, the muscle layers are closed with sutures and thoracotomy closure is completed. 
